# Evaluating the Diagnostic Accuracy of Point-of-Care CXCL13 Measurements for Lyme Neuroborreliosis

**DOI:** 10.3390/diagnostics16101424

**Published:** 2026-05-07

**Authors:** Lasse Fjordside, Mathilde Ørbæk, Thomas Bryrup, Louise Helvig Kaas, Alex Christian Yde Nielsen, Nikolai Søren Kirkby, Helene Mens, Anne-Mette Lebech

**Affiliations:** 1 Department of Infectious Diseases, Copenhagen University Hospital, Rigshospitalet, 2100 Copenhagen, Denmark; 2 Department of Clinical Microbiology, Copenhagen University Hospital, Rigshospitalet, 2100 Copenhagen, Denmark; 3 Department of Clinical Medicine, Faculty of Health and Medical Sciences, University of Copenhagen, 2200 Copenhagen, Denmark

**Keywords:** Lyme neuroborreliosis, biomarkers, CXCL13, diagnostics

## Abstract

**Background**: Measurements of the B-cell attracting chemokine CXCL13 in cerebrospinal fluid (CSF) has become increasingly popular in the diagnostic work-up for Lyme neuroborreliosis (LNB). ReaScan is a point-of-care test for CXCL13 with a short turnaround-time. However, the diagnostic cut-off value for LNB has not been firmly established. **Methods**: We conducted a retrospective cohort study including all ReaScan CXCL13 measurements performed on CSF samples at Copenhagen University Hospital, Rigshospitalet, from patients of all ages suspected for LNB in 2020–2025. Patients were categorized in accordance with the European Federation of Neurological Societies (EFNS) diagnostic guidelines for LNB as definite, possible, or non-LNB based on a retrospective review of patient records. The manufacturer-informed cut-off value for positive test results was applied and the diagnostic accuracy was calculated based on the ability to identify patients with possible and definite LNB. In addition, a Youden-index optimized cut-off value was calculated and evaluated on the cohort. **Results**: A total of 1181 ReaScan CXCL13 measurements from children and adults were evaluated against final diagnoses. Using the manufacturer-informed cut-off value (14 AU/mL) ReaScan CXCL13 reached a sensitivity of 38% and a specificity of 98% for identifying cases of possible and definite LNB. A Youden-index optimized cut-off value (1.79 AU/mL) significantly improved the diagnostic accuracy reaching a sensitivity value of 71% and a specificity of 92%. **Conclusions**: These real-world clinical data suggest that the current cut-off value for the ReaScan CXCL13 analysis is not optimal for diagnosing LNB. Lowering the cut-off level would significantly increase the diagnostic accuracy.

## 1. Introduction

Lyme neuroborreliosis (LNB) is a bacterial nervous system infection caused by tick-transmitted spirochetes of the *Borrelia burgdorferi* (*Bb*) sensu lato (s.l.) complex. LNB is endemic in many regions of the northern hemisphere where incidence rates vary between 3.2 and 7.8 per 100,000 person/year [1,2,3,4,5,6,7]. Current diagnostic modalities for LNB have several limitations that make diagnosing LNB challenging. Direct pathogen identification with PCR or cultivation has an exceedingly low yield [8,9,10,11,12,13,14,15] and *Bb*-specific antibodies appear late after infection and may remain elevated for several years after successful treatment [16,17]. In Europe, the currently used diagnostic criteria were established by The European Federation of Neurological Societies (EFNS) in 2010 and include the following three elements: (1) typical neurological symptoms, (2) elevated white-blood-cell count in CSF (CSF WBC), and (3) either a positive *Bb*-specific intrathecal antibody index test (*Bb* AI), a positive PCR, or positive cultivation [18]. The diagnosis is considered ‘definite’ if 3/3 criteria are fulfilled and ‘possible’ if 2/3 criteria are fulfilled. Over the last decade, a significant amount of evidence has confirmed the value of C-X-C motif ligand 13 (CXCL13) as a biomarker in CSF for LNB [19]. CXCL13 is a B-cell attracting chemokine that may be produced and secreted by several different immunological cell populations including macrophages, follicular dendritic cells, stroma cells, and subsets of T cells [20,21,22]. Elevated CXCL13 in CSF has demonstrated a high level of diagnostic accuracy for LNB and is thought to be especially valuable in early and treatment-naïve cases [19,23,24,25]. CXCL13 has a relatively high specificity for LNB but may be elevated in other conditions like neurosyphilis, CNS lymphoma, and multiple sclerosis [24,26,27,28]. The concentration of CXCL13 can be quantified with ELISA or semi-quantified using a lateral flow assay. ReaScan is a compact, lateral flow-based point of care (POC) platform designed for rapid, semi-quantitative CXCL13 measurement, eliminating the need for batch processing and enabling same-visit diagnostics. ReaScan translates the read value in arbitrary units per milliliter (AU/mL) to an interval of picograms per milliliter (pg/mL) that corresponds to the quantified ELISA derived values. Several studies have found a high degree of concordance between ReaScan values and ELISA results [29,30,31]. However, the currently used manufacturer-informed cut-off values for the ReaScan POC assay have not been firmly established in a clinical setting.

In this study, we aimed to evaluate the diagnostic accuracy of ReaScan CXCL13 for the diagnosis of LNB. We further aimed to explore whether the diagnostic accuracy could be improved by adjusting the cut-off value. As secondary outcomes, we wanted to investigate potential predictors of elevated CXCL13 including age, sex, symptom duration, pleocytosis, and antibiotic therapy prior to the diagnostic lumbar puncture.

## 2. Materials and Methods

### 2.1. Study Design, Study Population, and Sample Collection

We conducted a retrospective cohort study including all ReaScan CXCL13 measurements performed on CSF samples at the Department of Clinical Microbiology, Copenhagen University Hospital, Rigshospitalet, Copenhagen, Denmark, from patients of all ages suspected for LNB in 2020–2025. Patient samples originated from hospitals across the Region of Eastern Denmark (total population of ~2.8 million inhabitants). The region is considered endemic to Lyme borreliosis.

All available CXCL13 measurements performed in the study period were screened for inclusion. For consecutive samples from the same patient, only the first sample was included. Samples originating from hospitals outside the defined study region were excluded in accordance with the health judicial permissions of the study. Samples without available corresponding *Borrelia burgdorferi* intrathecal antibody index (*Bb* AI) test results were excluded in order to enable the classification of LNB in accordance with EFNS criteria and to minimize the risk of the inclusion of ‘off-label’ diagnostic use of CXCL13 in cases not suspected for LNB. Cases of LNB were classified in accordance with the established EFNS criteria [18]. Thus, patients with possible LNB fulfilled 2/3 criteria, patients with definite LNB fulfilled 3/3 criteria, and non-LNB controls were defined as fulfilling ≤1 of the 3 EFNS criteria. Pleocytosis was defined as >5 cells/µL. A Bb AI > 0.3 was considered positive. Final diagnoses of non-LNB patients were established through review of patient records ≥ 6 months after the CXCL13 analysis date.

### 2.2. Sample Analysis

All samples were analyzed directly after clinical collection at the Department of Clinical Microbiology at Copenhagen University Hospital, Rigshospitalet, on the rapid cassette-based immune chromatographic system, ReaScan CXCL13 lateral flow assay (Reagena, Toivala, Finland). Analyses were performed in accordance with the manufacturer’s instructions. Briefly, 100 µL of CSF was pipetted into the conjugate tube, mixed and transferred to the lateral flow cassette. After 20 min, the test cassette was read with the ReaScan reader providing values in AU/mL. Reads were then translated to semi-quantitative CXCL13 concentration intervals based on the manufacturer-informed cut-off values. Manufacturer-informed cut-off values varied across kits used in the study period from 11 to 17 AU/mL. Therefore, the median cut-off value across kits (14 AU/mL) was used in the analyses of diagnostic accuracy. All reads below 14 AU/mL were considered negative and all above were considered positive. According to the manufacturer-informed instructions, a read above this cut-off value would correspond to an ELISA value >250 pg/mL. Borrelia-specific intrathecal antibody index (*Bb* AI) was measured using a second-generation, flagella antigen-based capture enzyme immunoassay (IDEIA LNB, Oxoid, Hampshire, UK). 

### 2.3. Data Analysis

Data analyses were performed using RStudio (version 4.5.0) [32]. The diagnostic accuracy was assessed through calculation of sensitivity, specificity, positive predictive value, and negative predictive values and supplemented by receiver operating characteristic (ROC) curve and calculation of the area under the curve (AUC). The disease reference was possible and definite LNB combined and patients without LNB constituted the control-group. Optimization of the cut-off value in AU/mL was calculated using the Youden-index formula. Wilcoxon rank sum test and Spearman rank correlation test were used to calculate *p*-values for the difference in distributions across groups.

CXCL13 values were highly zero-inflated and right-skewed. Therefore, a two-step hurdle model was used to assess the effects of clinical covariates in patients with possible and definite LNB.

In the first step, logistic regression analyses were used to estimate odds ratios (ORs) for detectable CXCL13 (>0.0 AU/mL). Univariate analyses included age, age group, sex, CSF WBC, CSF protein, *Bb* serum IgG, symptom duration, and antibiotic therapy prior to diagnostic lumbar puncture. Covariates with significant ORs in univariate analyses were included in a multivariable logistic regression to estimate adjusted ORs and 95% confidence intervals (CIs).

In the second step, a gamma regression model was applied to assess any associations between the level of CXCL13 and the selected covariates. This analysis only included non-zero CXCL13 values in order to avoid false associations caused by the large amount of zero values.

Spearman rank correlation and Wilcoxon rank sum tests were used to evaluate inter-group comparisons of the distribution of CXCL13 values.

*p*-values < 0.05 were considered significant and 95% confidence intervals (CIs) were reported where indicated.

### 2.4. Risk of Bias Assessment

By restricting inclusion to patients with an available ReaScan CXCL13 result, we may have introduced a selection bias since some patients could have been evaluated for LNB without having the analysis of CXCL13 performed. However, according to local guidelines, ordering of the *Bb* AI test should automatically prompt ReaScan CXCL13 analysis and vice versa, which likely limits the extent of such bias. We also excluded cases without an available *Bb* AI test to enable classification according to the EFNS criteria. Although this approach may also introduce selection bias, inclusion of patients without *Bb* AI would preclude a meaningful assessment of diagnostic accuracy, as these cases could not be reliably categorized.

Partial incorporation bias is inevitable when evaluating the diagnostic accuracy of CXCL13 as a biomarker for LNB, because CSF WBC and *Bb* AI are essential diagnostic criteria and biologically associated with CXCL13.

### 2.5. Ethics

This study was conducted in accordance with the permission for clinical quality assessment provided by the Health Judicial Department at Copenhagen University Hospital, Rigshospitalet, Denmark (p-2024-17995).

## 3. Results

**Figure 1:**
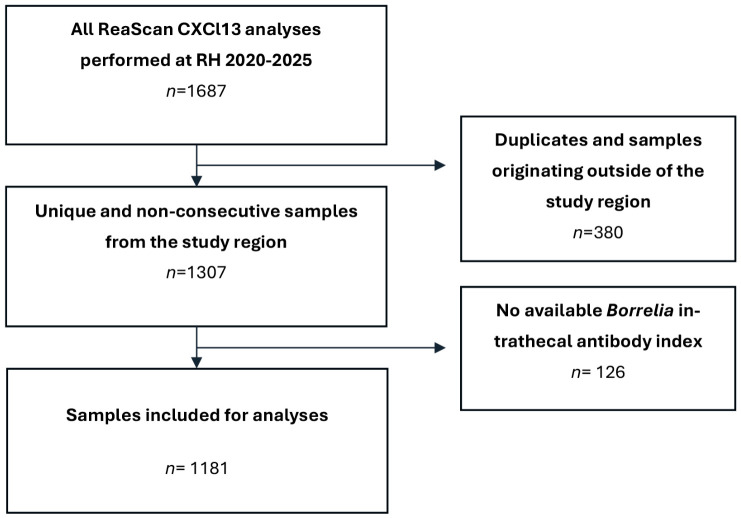
Establishment of the study cohort. Flowchart of in- and exclusion.

A total of 1687 ReaScan CXCL13 test results were screened for inclusion and 1181 were found eligible and included for the final analyses. A total of 380 test results were excluded because they were either consecutive samples from the same patient or originated from outside the region. An additional 126 test results were excluded because they lacked an available *Bb* AI (Figure 1). Baseline characteristics of included patients are available in Table 1. The non-LNB group included patients with a broad spectrum of diagnoses. An overview of non-LNB diagnostic sub-groups is available in the Supplementary Materials.

**Figure 2:**
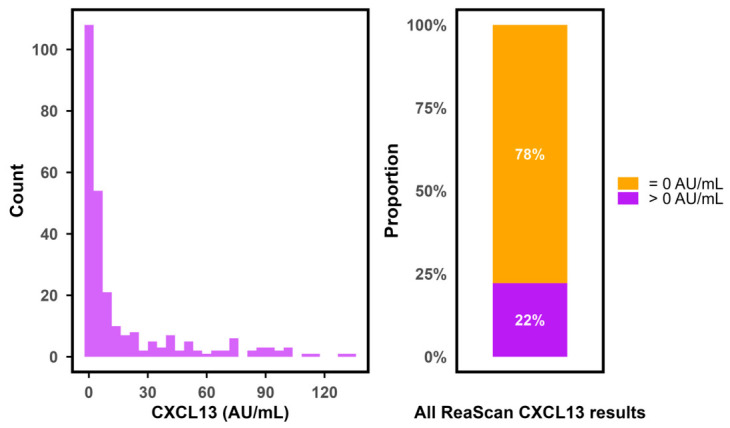
Distribution of ReaScan CXCL13 values across the entire study-population **Left side**: density barplot illustrating the distribution of all non-zero CXCL13 values in the cohort. **Right side**: stacked barplot illustrating the proportion of zero and non-zero values among all ReaScan CXCL13 results. CXCL13 = C-X-C motif ligand 13, AU/mL = arbitrary units per milliliter.

**Figure 3:**
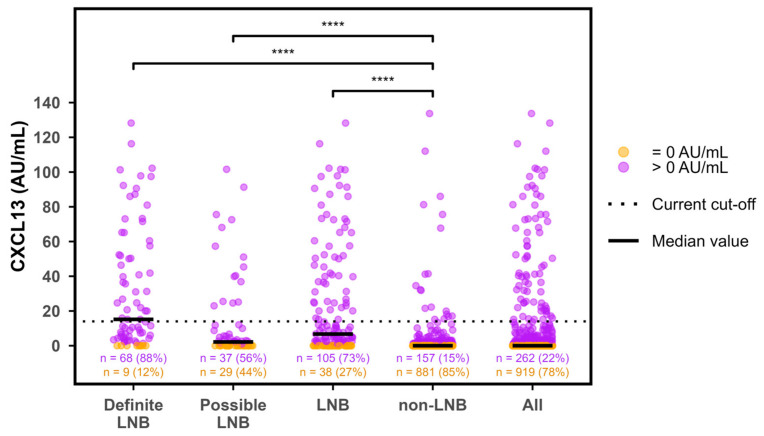
CXCL13 values by diagnostic group. LNB = Lyme neuroborreliosis, CXCL13 = C-X-C motif ligand 13, AU/mL = arbitrary units per milliliter. The ‘LNB’ group is definite and possible LNB combined. The ‘All’ group is all groups combined. Horizontal bars indicate median CXCL13 values. Dotted horizontal line indicates current cut-off value (14 AU/mL). Orange color indicates CXCL13 values equal to 0.0 AU/mL and purple color indicates values > 0.0 AU/mL. *p*-values calculated by Wilcoxon rank sum test are indicated on brackets with number of stars indicating ordinate categories: **** = *p* < 0.001.

**Figure 4:**
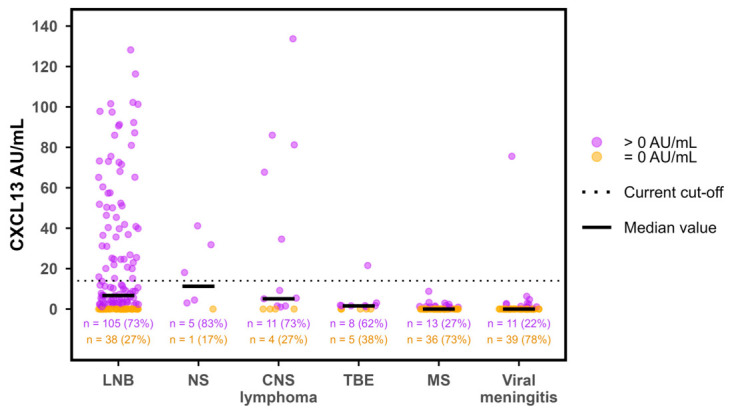
Comparison of CXCL13 values in LNB and non-LNB diagnostic groups. Distribution of CXCL13 values in LNB (possible and definite combined), the three non-LNB diagnoses with *n* > 5 and the highest proportion of non-zero CXCL13 values (Neurosyphilis, CNS lymphoma, and TBE), and MS and viral meningitis. LNB = Lyme neuroborreliosis, CNS = Central nervous system, TBE = Tick-borne encephalitis, MS = Multiple sclerosis, CXCL13 = C-X-C motif ligand 13, and AU/mL = arbitrary units per milliliter. Horizontal bars indicate median CXCL13 values. Dotted horizontal line indicates current cut-off value (14 AU/mL). Orange color indicates CXCL13 values equal to 0.0 AU/mL and purple color indicates CXCL13 values > 0.0 AU/mL.

**Figure 5:**
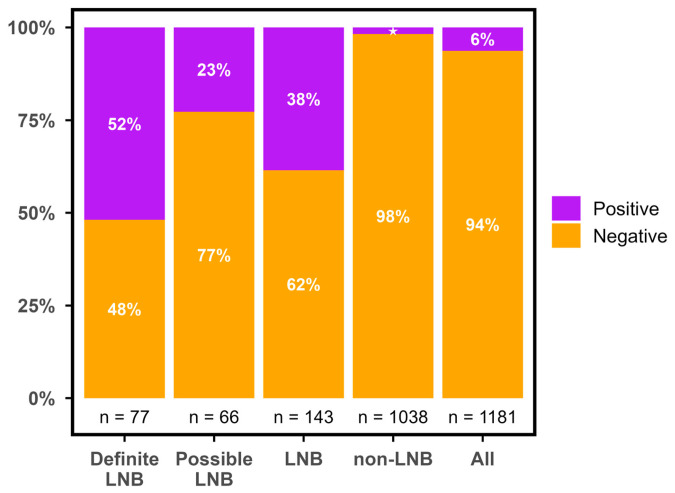
Proportions of positive and negative ReaScan CXCL13 results by diagnostic group using the median manufacturer-informed cut-off value for the study period (14 AU/mL). LNB = Lyme neuroborreliosis. This group is definite LNB and possible LNB combined, CXCL13 = C-X-C motif ligand 13, and AU/mL = arbitrary units per milliliter. The ‘LNB’ group is possible and definite LNB combined. The ‘All’ group is all groups combined. ★ = 1.8%.

In the majority of samples, CXCL13 was undetectable on the ReaScan assay. Thus, only 262/1181 (22%) of the test results had a value above 0.0 AU/mL. Furthermore, the distribution of non-zero values was heavily right-skewed with a marked overweight in low values (Figure 2). Median CXCL13 values were significantly higher in patients with LNB compared to non-LNB (*p* < 0.0001) and higher in definite LNB compared to possible LNB (*p* < 0.001) (Figure 3). The primary non-LNB diagnostic groups with significantly elevated CXCL13 values were neurosyphilis (median 11 AU/mL), CNS lymphoma (median 5 AU/mL), and TBE (median 2 AU/mL) (Figure 4). However, elevated CXCL13 was also found in a variety of other diagnostic groups including encephalitis, bacterial CNS infections, amyotrophic lateral sclerosis (ALS), multiple sclerosis (MS), and malignant CNS diseases. Using the current manufacturer-informed cut-off value (median in 2020–2025: 14 AU/mL), a total of 38.5% of patients with LNB and 1.8% of non-LNB patients had a positive ReaScan CXCL13 test (Figure 5).

**Figure 6:**
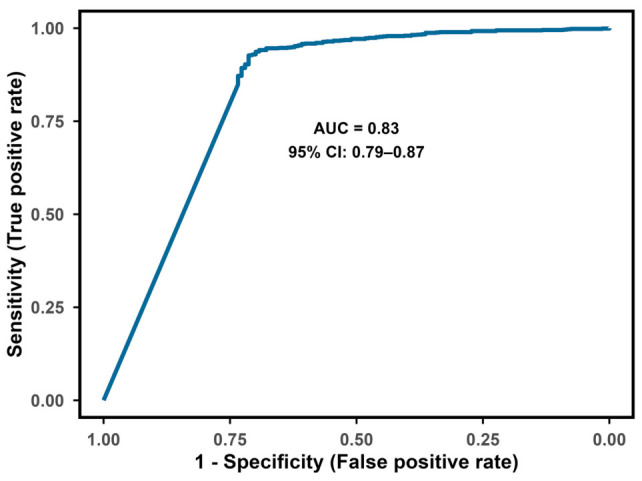
Receiver operating characteristic (ROC) curve and area under the curve (AUC) for the ability of ReaScan CXCL13 to identify patients with LNB (possible + definite). AUC = Area under the curve. CI = Confidence interval.

**Figure 7:**
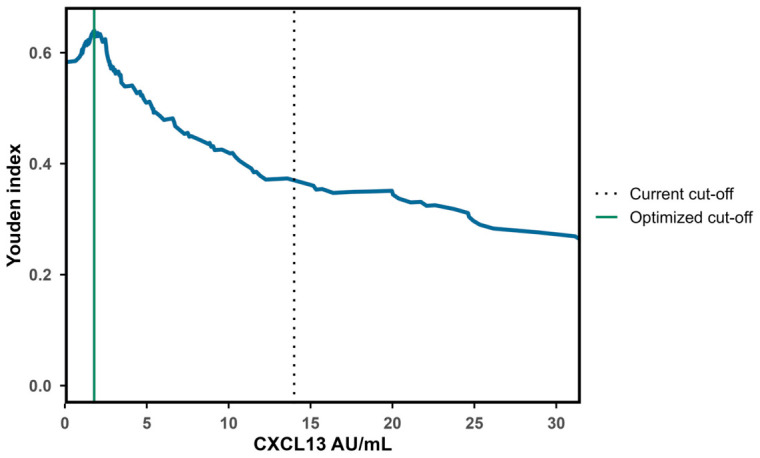
Youden indices by CXCL13 values. Identification of optimized cut-off value. Youden index = (sensitivity + specificity) − 1. CXCL13 = C-X-C motif ligand 13, AU/mL = arbitrary units per milliliter. Dotted gray vertical line indicates current cut-off value (14 AU/mL) and solid green vertical line indicates optimized cut-off value (1.79 AU/mL). X-axis only includes CXCL13 values from 0 to 30 AU/mL for better visualization of cut-off values and change in Youden indices. Abbreviations: CXCL13 = C-X-C motif ligand 13; AU/mL = arbitrary units per milliliter. Youden index = (sensitivity + specificity) − 1.

**Figure 8:**
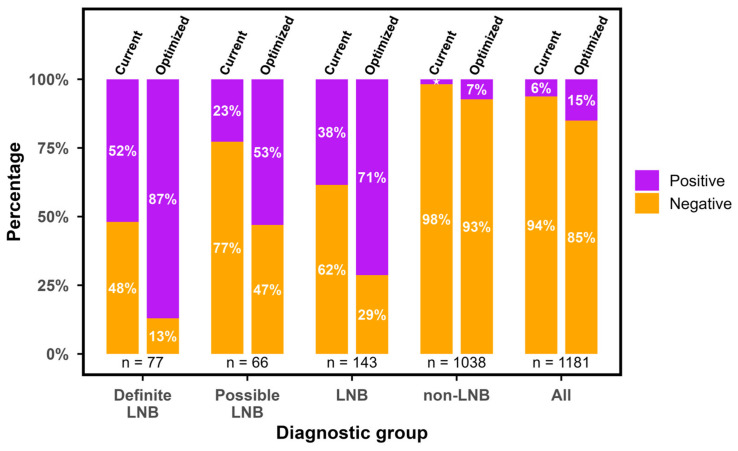
Comparison of the distribution of CXCL13 test results using the current and the optimized cut-off value. LNB = Lyme neuroborreliosis. This group is definite LNB and possible LNB combined, CXCL13 = C-X-C motif ligand 13, and AU/mL = arbitrary units per milliliter. The ‘LNB’ group is possible and definite LNB combined. The ‘All’ group is all groups combined. ★ = 1.8%.

With possible and definite LNB as cases and non-LNBs as reference, ReaScan CXCL13 reached an overall AUC of 0.83 (CI = 0.79–0.87) using the numeric values of test results (Figure 6). Applying the manufacturer-informed cut-off value (14 AU/mL), ReaScan CXCL13 reached a sensitivity of 38% and a specificity of 98% for identifying cases of possible and definite LNB (Table 2). The Youden-index optimized cut-off value was 1.79 AU/mL providing a sensitivity value of 71% and a specificity of 92% (Table 2 and Figure 7 and Figure 8). Due to the low disease prevalence, the relatively small reduction in specificity caused a significant reduction in the positive predictive value (PPV) (74% to 57%), while the negative predictive value only increased slightly (92% to 96%). To highlight the significant effect of disease prevalence on predictive values, we calculated PPVs and NPVs for different hypothetical levels of disease prevalence (Supplementary Table S1). Noticeably, the positive likelihood ratio (LR+) decreased from 21.01 to 9.74 and the negative likelihood ratio (LR−) dropped from 0.63 to 0.31 when applying the optimized cut-off value. Thus, using the manufacturer-informed cut-off, a positive test resulted in a 21-times increased likelihood of LNB and a negative test reduced the likelihood to 37%. With the optimized cut-off, a positive test is associated with an approximately 10-times higher likelihood for LNB and a negative test reduces the likelihood of LNB to 69% (Table 2). The proportion of LNB patients with a positive CXCL13 test increased from 38% to 71% with the optimized cut-off and the proportion of non-LNB patients with a negative test result increased from 2% to 7% (Table 2). To evaluate the impact of variation in applied cut-off values during the study period, a sensitivity analysis was performed using the lowest and highest thresholds (11 and 17 AU/mL). Sensitivity ranged from 42% (11 AU/mL) to 36% (17 AU/mL) (Supplementary Table S2).

We additionally calculated diagnostic accuracies for the following constitutions of case groups: (1) only definite LNB, (2) definite LNB and possible LNB with pleocytosis (>5 cells/µL), and (3) definite LNB and possible LNB without pleocytosis but positive *Bb* AI. Only including definite LNB increased sensitivity to 52%, and sensitivity was markedly higher when including the subgroup of patients classified as possible LNB with a positive *Bb* AI than those with pleocytosis (49% vs. 39%). Results are available in Supplementary Table S3.

In a multivariate logistic regression model including age, sex, CSF WBC, CSF protein, symptom duration, and antibiotic therapy prior to diagnostic lumbar puncture, only CSF protein remained significantly associated with CXCL13 detectability. However, the association with CSF WBC was likely masked by collinearity with CSF protein.

In the gamma regression model assessing CXCL13 levels among patients with non-zero values, CSF protein remained the only significant covariate after adjustment. When CSF protein was removed from the model, CSF WBC became significantly associated, suggesting collinearity between the two variables. Coefficients, confidence intervals and *p*-values for both regression models are available in Supplementary Table S4A,B.

Spearman correlation showed a weak but significant inverse association between CSF WBC and symptom duration (coefficient: −0.177, *p* = 0.035). However, symptom duration showed no significant association with CXCL13 detectability or level in the hurdle model.

No statistically significant differences in CXCL13 values were found by groupwise comparisons of LNB patients with short (<14 days), intermediate (14–28 days), and long (>28 days) symptom duration (Supplementary Figure S1a). Similarly, no significant differences in CXCL13 levels were found between patients who received antibiotics prior to lumbar puncture and those who did not (Supplementary Figure S1b).

Across all models, CSF protein was the strongest determinant of CXCL13 detectability and levels (Supplementary Figure S1c,d).

Among patients with CXCL13 values >0.0 (*n* = 262), a total of 47 (18%) had a CSF WBC <5. In this group, a total of 23 patients had a final diagnosis that did not rule out potential missed early LNB (Bell’s palsy *n* = 4 and unknown *n* = 19).

## 4. Discussion

To our knowledge, this is the largest study assessing the diagnostic accuracy of ReaScan CXCL13 for LNB to date. It is also the first clinical evaluation performed on real-world data and a largely unselected patient cohort. We found a markedly lower sensitivity than previous studies even after optimizing the cut-off value. CXCL13 levels were not associated with symptom duration in patients with LNB, but correlated with markers of central nervous system inflammation, including cerebrospinal fluid leukocyte count and protein concentration. In a diagnostic framework, ReaScan CXCL13 appears best positioned as an adjunctive rule-in test—particularly in early disease phases or diagnostically ambiguous cases.

Three studies have previously evaluated the diagnostic accuracy of ReaScan CXCL13 in LNB.

In 2018, Pietikäinen et al. compared ReaScan and ELISA CXCL13 measurements on CSF samples from 220 patients with LNB and other conditions [29]. Analyses of diagnostic accuracy were performed on 94 out of 220 patients with available diagnoses including 13 patients with LNB. Authors found a sensitivity value of 100% and a specificity of 96% for definite LNB [29], using the manufacturer indicated cut-off at that time of 30 AU/mL as cut-off.

In 2020, Ziegler et al. compared ReaScan with the Euroimmun ELISA CXCL13 assay on CSF from a total of 90 patients (34 with definite LNB, 10 with possible LNB, and 46 with other inflammatory CNS conditions) [30]. Authors reported a sensitivity value of 100% and a specificity of 84.8% for the ELISA assay using a cut-off value of 78.6 pg/mL. Using an optimized cut-off value (22.5 AU/mL), ReaScan CXCL13 reached a sensitivity of value 91.2% and a specificity of 93.5% for definite LNB.

In 2022, Haglund et al. analyzed a total of 209 CSF samples from patients defined as definite and non-LNB in accordance with EFNS guidelines, and a group named probable early LNB consisting of patients with typical symptoms of LNB, short symptom duration, and pleocytosis [31]. Authors reported a sensitivity of 78% and a specificity of 95% for definite and probable LNB. Manufacturer-informed cut-offs were used, but the exact value in AU/mL was not reported.

Notably, two of the three studies (Pietikäinen et al. [29] and Ziegler et al. [30]) reported significantly higher diagnostic sensitivities than what we found. There are likely several different reasons for this discrepancy. The retrospective case–control design with low degrees of heterogeneity in both case and control groups will tend to inflate diagnostic accuracy. Thus, by only including patients with definite LNB as cases and using few different diagnostic groups for comparison will likely result in a significantly higher diagnostic accuracy than what would be expected in a real clinical context. Noticeably, only Haglund et al. included patients with possible LNB when calculating diagnostic accuracy. This most likely explained the significantly lower sensitivity reported in this study, which was largely comparable to what we found (78% vs. 71%).

Of notice, Pietikäinen et al. [29] and Ziegler et al. [30] used significantly higher cut-off values than both the original and the optimized cut-off values in our study (30 and 22.5 AU/mL vs. 14 AU/mL). The differences in numeric cut-off values likely reflect kit-specific variations.

ReaScan CXCL13 results are reported in arbitrary units and are therefore not directly comparable across studies. Consequently, the optimized cut-off identified in this study (1.79 AU) cannot be transferred to other settings or externally validated in a meaningful way. Nonetheless, the substantially lower optimal threshold suggests that the manufacturer-recommended cut-off is likely too high for diagnosing Lyme neuroborreliosis (LNB). This is consistent with the corresponding ELISA-equivalent threshold (>250 pg/mL), which exceeds the commonly reported optimal range for ELISA assays (approximately 50–100 pg/mL) [19,25,30]. The relatively low sensitivity (71%), despite an optimized cut-off value relatively close to zero and even when restricting cases to definite LNB, could suggest that the lower level of detection for ReaScan CXCL13 is too high. Benchmarking studies with ELISA have been performed, but only in the previously described case–control settings. To answer this question, future studies should include a broad spectrum of cases and controls mimicking the real-world variation and compare ReaScan results with ELISA.

We were not able to find any statistically significant association between symptom duration and CXCL13 in our data. This is interesting because CXCL13 is generally considered a marker of early LNB [19,23,24,33,34,35]. It is however consistent with the findings of several previous studies [26,36,37]. Thus, CXCL13 should probably rather be considered an early marker of LNB than a marker of early LNB. Surprisingly, antibiotic therapy prior to lumbar puncture was not associated with lower CXCL13 values. However, a modified time- and/or dose-dependent variable would probably have been more physiologically meaningful and could potentially have yielded a result in line with previous findings [26]. CSF protein levels were strongly correlated with CXCL13 detectability and level in patients with LNB. CSF WBC showed a similar trend across analyses, though the effect was smaller and partially masked by collinearity with CSF protein. This is in line with previous findings [24,36,37,38]. Interestingly, we also found a weak but significant invert association between CSF WBC and symptom duration. CSF WBC could therefore potentially have confounded results in previous studies leading to the false conclusion that CXCL13 is associated with early LNB.

### Strengths and Limitations

Evaluating the diagnostic performance on real-world data and including both possible and definite LNB is a particular strength of this study, providing a more clinically meaningful assessment of the diagnostic accuracy. The large sample size and the unselected patient cohort are also a significant strength.

Due to the retrospective design, we had to rely on the clinical information available in medical records. As in any study aiming to assess diagnostic accuracies in real-world settings, the risk of misclassification is essential to acknowledge. We performed reviews of patient medical records to minimize this risk. Thus, apart from fulfilling the EFNS criteria, we ensured that all patients in both LNB categories (definite and possible) in fact had LNB as their final diagnosis and explained the clinical picture they presented upon admission.

We used the median cut-off value across all kits in the study period to evaluate the diagnostic accuracy. While using kit-specific cut-off values would have been ideal, we performed a sensitivity analysis that showed a very limited effect across all metrics of diagnostic accuracy when applying the highest and lowest values of the period (Supplementary Table S2).

We chose to use the Youden index to calculate the optimized cut-off value because it balances sensitivity and specificity. However, one could argue that if the place of CXCL13 in the diagnostic work-up for LNB is primarily as a confirmatory test in patients with relevant symptoms and pleocytosis, then maximizing specificity would be more appropriate. However, as shown in the main analysis, maximizing specificity dramatically reduces sensitivity to a level that markedly affects the clinical utility because of the small proportion of LNB patients with a positive test.

## 5. Conclusions

In this large real-world evaluation of diagnostic accuracy, ReaScan CXCL13 demonstrated high specificity but poor sensitivity for diagnosing LNB when applying the manufacturer-informed cut-off. Lowering the cut-off substantially improved sensitivity, albeit with a modest reduction in specificity, and suggests that the manufacturer-defined threshold is likely too high for use in routine clinical settings. However, even at the optimized cut-off, sensitivity remained limited.

These findings indicate that ReaScan CXCL13 may have value primarily as a rule-in test, where a positive result substantially increases the likelihood of LNB, but has insufficient sensitivity to be used as a rule-out test.

Future studies should include head-to-head comparisons with ELISA-based CXCL13 measurements in unselected, real-world populations to determine whether the analytical sensitivity of the ReaScan assay is a limiting factor.

## Figures and Tables

**Table 1:** Baseline characteristics of included patients.

Characteristic	Definite LNB *n* = 77	Possible LNB *n* = 66	LNB ^1^ *n* = 143	Non-LNB *n* = 1038	All *n* = 1181
**Female sex**	35 (45%)	28 (42%)	63 (44%)	530 (51%)	593 (50%)
**Age in years**	52 (38–66)	50 (30–63)	51 (32–65)	48 (30–63)	48 (30–63)
**Age group**					
Child	8 (10%)	12 (18%)	20 (14%)	119 (11%)	139 (12%)
Adult	69 (90%)	54 (82%)	123 (86%)	919 (89%)	1042 (88%)
**Department** ^2^					
Neurology	26 (34%)	25 (38%)	51 (36%)	566 (55%)	617 (52%)
Infectious Diseases	39 (51%)	25 (38%)	64 (45%)	270 (26%)	334 (28%)
Pediatrics	7 (9.1%)	11 (17%)	18 (13%)	84 (8.1%)	102 (8.6%)
Other	5 (6.5%)	5 (7.6%)	10 (7.0%)	118 (11%)	128 (11%)
**CXCL13 Categoric**					
Positive	40 (52%)	15 (23%)	55 (38%)	19 (1.8%)	74 (6.3%)
Negative	37 (48%)	51 (77%)	88 (62%)	1019 (98%)	1107 (94%)
**CXCL13 AU/mL**	15 (4–52)	2 (0–12)	7 (0–40)	0 (0–0)	0 (0–0)
**CSF WBC** × 10^6^ cells/L	122 (64–224)	66 (15–138)	90 (37–208)	3 (3–8)	3 (3–19)
**CSF Protein** g/L	1.18 (0.67–1.96)	0.58 (0.40–0.87)	0.77 (0.47–1.41)	0.40 (0.30–0.59)	0.43 (0.31–0.64)
***Bb* serum IgG**					
Negative	3 (6.3%)	22 (47%)	25 (26%)	730 (83%)	755 (78%)
Positive	45 (94%)	25 (53%)	70 (74%)	148 (17%)	218 (22%)
***Bb* AI**					
Positive	77 (100%)	12 (18%)	89 (62%)	5 (0.5%)	94 (8.0%)
Inconclusive	0 (0%)	6 (9.1%)	6 (4.2%)	26 (2.5%)	32 (2.7%)
Negative	0 (0%)	48 (73%)	48 (34%)	1007 (97%)	1055 (89%)

^1^: ‘LNB’ is possible and definite LNB combined. ^2^: Department from where the CXCL13 analyses were requested. LNB = Lyme neuroborreliosis, CSF = cerebrospinal fluid, CXCL13 = C-X-C motif ligand 13, AU/mL = arbitrary units per milliliter, WBC = white-blood-cell count, ×10^6^ cells/L. CSF protein, g/L. *Bb* = *Borrelia burgdorferi*. AI = intrathecal antibody index. Quantifications: Quantitative variables are reported with median and interquartile range (IQR). Categorical variables are reported with number (*n*) and proportion (%). CXCL13 ReaScan indicates positive and negative results using the manufacturer-informed cut-off value (14 AU/mL (median for the study period)).

**Table 2:** Diagnostic accuracy metrics comparing the current and optimized cut-off value.

Diagnostic Metric	Original Cut-Off	Optimized Cut-Off	Delta
Accuracy	91%	90%	−1
Sensitivity	38%	71%	+33
Specificity	98%	92%	−6
PPV	74%	57%	−17
NPV	92%	96%	+4
LR+	21.01	9.74	−11.27
LR−	0.63	0.31	−0.32
Kappa	0.46	0.58	+0.12
Balanced accuracy	68%	82%	+14

PPV = positive predictive value (True Positives/(True Positives + False Positives), NPV, Metric definitions: Accuracy = (True Positives + True Negatives)/(True Positives + True Negatives + False Positives + False Negatives), Sensitivity = True Positives/(True Positives + False Negatives), Specificity = True Negatives/(True Negatives + False Positives), PPV = True Positives/(True Positives + False Positives), NPV = True Negatives/(True Negatives + False Negatives), Positive Likelihood Ratio (LR+) = sensitivity/1 − specificity, Negative Likelihood Ratio (LR−) = 1 − sensitivity/specificity, Cohen’s Kappa = (Po − Pe)/(1 − Pe) where Po is the observed agreement Po = (True Positives + True Negatives)/total N) and Pe is the expected agreement Pe = ((TP + FN)/N) ∗ ((TP + FP)/N) + ((FP + TN)/N) ∗ ((FN + TN)/N) (TP = True positives, FP = False positives, TN = True negatives, FN = False Negatives and N = total number of individuals. Balanced accuracy = (sensitivity + specificity)/2.

## Data Availability

Full code script is available upon request. Raw data are protected by regulations on the distribution of personal data and will therefore not be available for external researchers.

## Supplementary Materials

The following supporting information can be downloaded at: https://www.mdpi.com/article/10.3390/diagnostics16101424/s1, Figure S1: Predictors of CXCL13 level and detectability in patients with LNB; Table S1: Positive- and negative predictive values calculated for different hypothetical prevalence-levels; Table S2. Diagnostic accuracy metrics of extreme cut-off values (11 and 17 AU) for ReaScan CXCL13; Table S3. Diagnostic accuracy metrics for case groups consisting of: (I) Definite LNB only (II) Definite LNB and Possible LNB with pleocytosis, but negative Bb AI, and (III) Definite LNB and Possible LNB without pleocytosis, but positive Bb AI; Table S4. Coefficients, confidence intervals, and *p*-values for the regression models.
