# Supplementary material for: Evaluating the Diagnostic Accuracy of Point-of-Care CXCL13 Measurements for Lyme Neuroborreliosis
Source: Diagnostics (Basel). 2026 May 7;16(10):1424. doi: 10.3390/diagnostics16101424 (PMC13205142; doi:10.3390/diagnostics16101424)
Supplement: Supplementary file 1 [file diagnostics-16-01424-s001.zip › diagnostics-4214386-supplementary.pdf]

Evaluating the diagnostic accuracy of point-of-care CXCL13 measurements for Lyme neuroborreliosis

Lasse Fjordside, Mathilde Ørbæk, Thomas Bryrup, Louise Helvig Kaas, Alex Christian Yde Nielsen, Nikolai Søren Kirkby, Helene Mens, Anne-Mette Lebech

Supplementary

Baseline characteristics

The final study-population included 12% children and 88% adults. Fifty percent of the study-population were female. CXCL13 analyses were ordered by clinical departments of neurology (n=617, 52%), infectious diseases (n=334, 28%), paediatrics (n=102, 8.6%), and other departments (n=128, 11%). A total of 143/1181 patients (12%) had a confirmed final diagnosis of LNB. Patients with LNB fulfilled ENFS criteria for possible LNB (n=66, 46%) or definite LNB (n= 77, 56%). Positive ReaScan CXCL13 test results using the current cut-off value (14 AU/mL) were found in 40/77 (52%) patients with definite LNB, in 15/66 (23%) patients with possible LNB and in 19/1181 (1.8%) patients without LNB. Median CXCL13 values varied across diagnostic groups from 15 AU/mL (IQR: 2,52) for patients with definite LNB, 2 AU/mL (IQR: 0,12) for patients with possible LNB and 0 AU/mL (IQR: 0,0) for patients without LNB. Median CSF WBC was highest in patients with definite LNB (122 x10^6 cells/L (IQR: 64, 224)), lower in patients with possible LNB (66 x10^6 cells/L (IQR: 15, 138)) and lowest in patients without LNB (3 x10^6 cells/L (IQR: 3, 8)). Mean CSF protein levels were also highest in patients with definite LNB (Median:1.18 (IQR: 0.67, 1.96)), lower in patients with possible LNB (Median: 0.58 (IQR: 0.40, 0.87)) and lowest in patients without LNB (Median: 0.40 (IQR: 0.30, 0.59)). All patients with definite LNB had a positive *Bb* AI. A total of 12/66 patients (18%) with possible LNB had a positive *Bb* AI, whereas this was the case for 5/1038 (0.5%) patients without LNB (Table 1).

Non-LNB diagnostic sub-groups

The remaining 1038 patients without LNB received final diagnoses within the following categories: neuropathies (n=133, 13%), idiopathic facial nerve palsy (n=99, 9.5%), multiple sclerosis (n=50, 4.8%), viral meningitis (n=50, 4.8%), encephalitis (n=46, 4.4%), Lyme borreliosis (n=39, 3.8%), dementia (n=28, 2.7%), musculoskeletal condition (n=25, 2.4%), vascular CNS disease (n=24, 2.3%), malignant CNS disease (n=22, 2.1%), CNS lymphoma (n=16, 1.5%), Tick-borne-encephalitis (TBE) (n=13, 1.3%), amyotrophic lateral sclerosis (ALS) (n=11, 1.1%), epilepsy or seizure (n=10, 1%), rheumatological disease (n=10, 1%), bacterial CNS infection (n=9, 0.9%), transverse myelitis (n=7, 0.7%), neurosyphilis (n=6, 0.6%), other (n=93, 9%), and 347 (33.4%) did not receive any final diagnosis.

Secondary outcome analyses

The secondary aim of this study was to assess potential predictors of elevated CXCL13. Due to the zero-inflated and heavily right-skewed data we chose to evaluate the potential effects of covariates on CXCL13 in two steps. First we used uni- and multivariate logistic regression models to look for associations between covariates and detectable CXCL13 defined as CXCL13 >0.0 AU/mL. To do this, we changed the CXCL13 variable from continuous to binary, consisting only of > or = 0.0 AU/mL values. This first step would answer if any of the included covariates were associated with an increased chance (odds ratio (OR)) of detectable CXCL13. In the second step, we only included CXCL13 values >0.0 AU/mL and included the same covariates as in the first step in a multivariate gamma-regression model to look for associations between covariates and changes in CXCL13 values. The following covariates were included in the regression analyses: age, sex, CSF WBC, CSF protein, , symptom duration, and antibiotic therapy prior to diagnostic lumbar puncture.

Table S1: Positive- and negative predictive values calculated for different hypothetical prevalence-levels.

Metric	Prevalence of LNB				
	**1%**	**5%**	**10%**	**12%**	**30%**
**PPV**	17.5%	52.5%	70%	74%	90%
**NPV**	99.4%	96.8%	93.5%	92%	78.8%

Sensitivity analysis

The kit-specific cut-off values for a positive test result varied during the study period from 11-17 AU. The median value (14 AU) for the period was therefore chosen as the basis for calculating diagnostic accuracy metrics in the main analyses. To assess the potential variation in diagnostic accuracy across the spectrum of cut-off values in the study-period, we performed a sensitivity analysis by calculating diagnostic accuracies using the extreme values of the interval (11 and 17AU) (Table S2).

Table S2. Diagnostic accuracy metrics of extreme cut-off values (11 and 17 AU) for ReaScan CXCL13

	11 AU	14 AU	17 AU
**Sensitivity**	42%	38.5%	36.4%
**Specificity**	97.9%	98.2%	98.5%
**PPV**	73.2%	74.3%	76.5%
**NPV**	92.4%	92.1%	91.8%
**Accuracy**	91.1%	90.9%	90.9%
**Balanced Accuracy**	69.9%	68.3%	67.4%
**Cohen's Kappa**	0.49	0.46	0.45

As illustrated in Table S2, shifting the cut-off value from the highest to the lowest extreme of the study-period, only causes minor variations in the parameters of diagnostic accuracy.

Table S3. Diagnostic accuracy metrics for case groups consisting of: I) Definite LNB only II) Definite LNB and Possible LNB with pleocytosis, but negative *Bb* AI), and III) Definite LNB and Possible LNB without pleocytosis, but positive *Bb* AI.

**Metric**	**Definite only (n=77)**	**Definite + possible with pleocyto-sis (n=77+139)**	**Definite + possible with positive AI (n=77+4)**
**Accuracy**	94%	91.3%	93.6%
**Sensitivity**	51.9%	39.6%	49.4%
**Specificity**	96.9%	98.2%	96.9%
**PPV**	54.1%	74.3%	54.1%
**NPV**	96.7%	92.4%	96.3%
**Cohen's Kappa**	0.5	0.47	0.48
**Balanced Accu-racy**	74.4%	68.9%	73.1%

Sensitivity was significantly higher when exclusively including patients with definite LNB as cases (52% compared with 38% in the main analysis including the complete population of definite and possible LNB combined). Comparison of the two subgroups of possible LNB, showed clearly higher sensitivity when only including the small subgroup of patients with positive AI in the group classified as possible LNB (49.4% vs 39.6%). However, the low number of patients with positive AI in the possible LNB group makes their effect on the diagnostic metrics marginal.

Table S4. Coefficients, confidence intervals, and p-values for the regression models

A. multivariate logistic regression model: CXCL13 >0.0 ~ age + age-group + sex + CSF WBC + CSF protein + symptom duration + antibiotic therapy prior to diagnostic lumbar puncture.

Variable	OR (95% CI)	*p*-value
Age (years)	0.99 (0.97–1.01)	0.229
Male sex	0.99 (0.38–2.6)	0.989
Symptom duration (days)	1 (0.99–1)	0.319
Antibiotics before LP	0.62 (0.21–1.8)	0.374
CSF WBC	1.01 (1–1.01)	0.133
CSF protein (per 0.1g/L)	1.3 (1.12–1.56)	0.00177

Values are odds ratios (OR) with 95% confidence intervals.

B. multivariate gamma-regression model: CXCL13 ~ age + age-group + sex + CSF WBC + CSF protein + symptom duration + antibiotic therapy prior to diagnostic lumbar puncture.

Variable	Ratio of means (95% CI)	*p*-value
Age (years)	1 (0.99–1.01)	0.907
Male sex	1.39 (0.93–2.07)	0.105
Symptom duration (days)	1 (1–1)	0.174
Antibiotics before LP	0.76 (0.47–1.28)	0.278
CSF WBC	1 (1–1)	0.717
CSF protein (per 0.1g/L)	1.09 (1.05–1.13)	<0.0001

Values are exponentiated coefficients (ratio of means) with 95% confidence intervals.

As indicated, only CSF protein remained significantly associated with odds of detectable CXCL13 in the multivariate logistic regression model and associated with level of CXCL13 in the gamma-regression model. However, when CSF protein was removed from the model, the association of CSF WBC with detectability and level of CXCL13 was significant, strongly suggesting collinearity. CSF WBC was also significantly associated with level of log(CXCL13) and when categorized, Wilcoxon rank sum tests confirmed the correlation of increasing levels of CSF WBC with increasing CXCL13 (Figure S1 C).

FigureS1. Predictors of CXCL13 level and detectability in patients with LNB.

a) CXCL13 values in LNB patients by symptom duration categories.

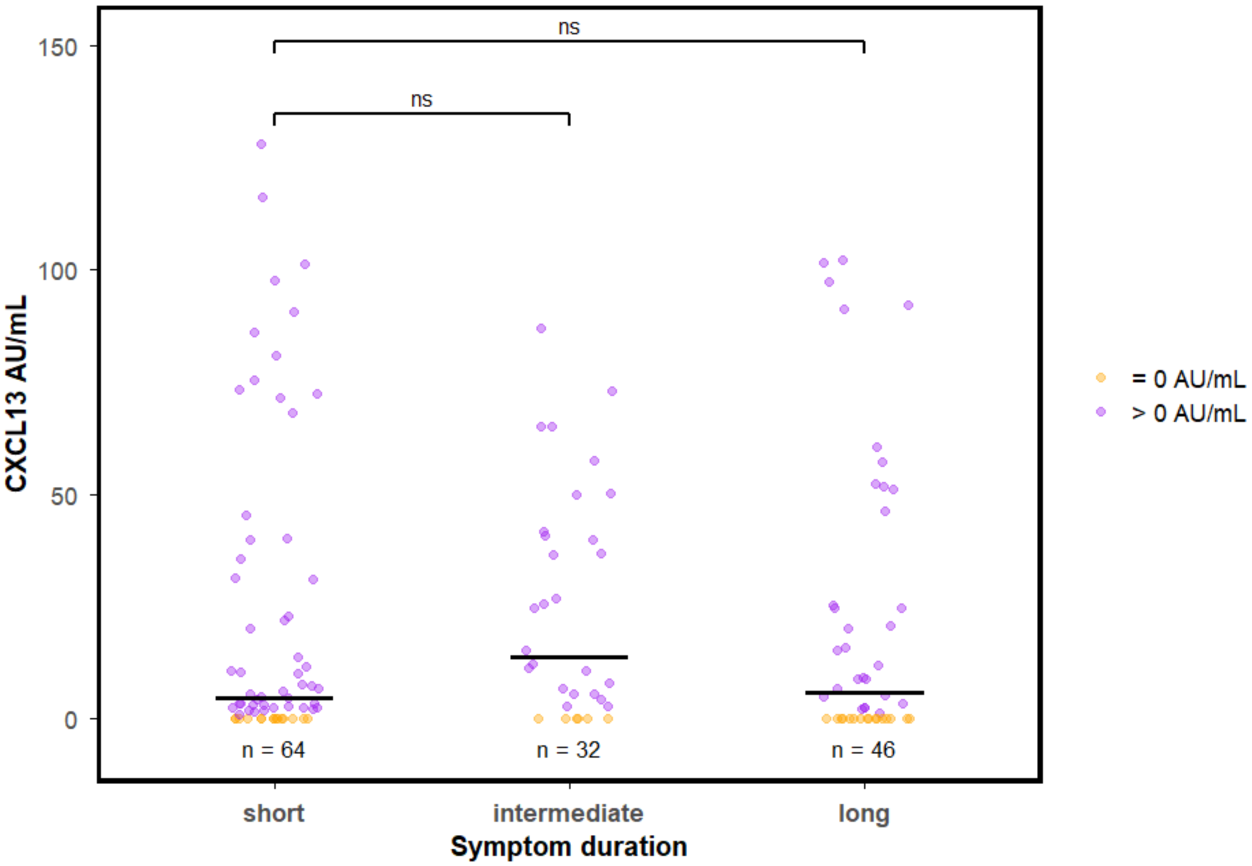

CXCL13= C-X-C motif ligand 13, AU/mL = arbitrary units per millilitre. Short symptom duration ≡ <14 days, Intermediate symptom duration ≡ 14 to 28 days, and Long symptom duration ≡ >28 days. Orange color indicates CXCL13 values equal to 0.0 AU/mL and purple color indicates values > 0.0 AU/mL Categoric significance levels of group comparisons are indicated on brackets (ns= not significant (p>0.05),*= p <0.05, **= p<0.01, ***=p<0.005, ****=p<0.001). Horizontal bars indicate median values for each group.

b) CXCL13 values in patients with LNB with and without antibiotic treatment prior to diagnostic lumbar puncture.

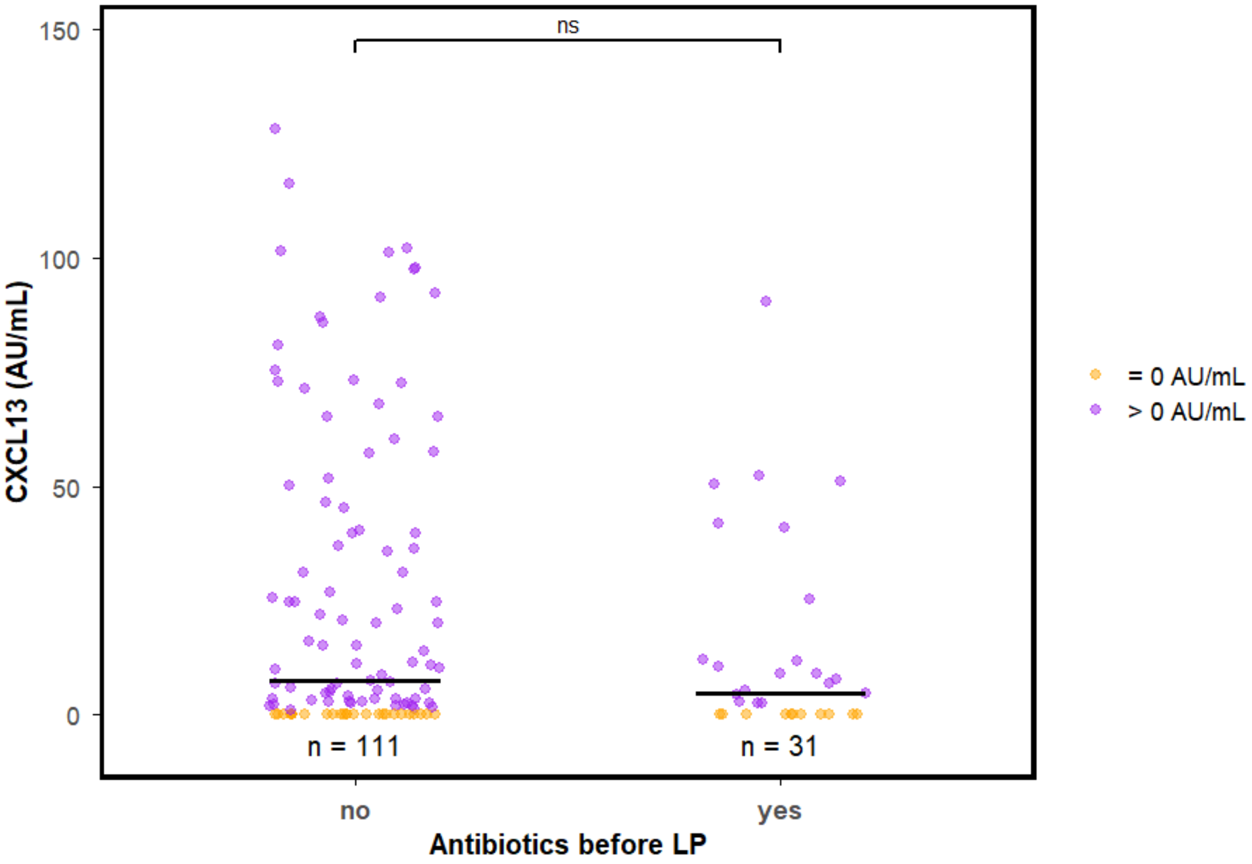

LP= Lumbar puncture, CXCL13= C-X-C motif ligand 13, AU/mL = arbitrary units per milliliter. Orange color indicates CXCL13 values equal to 0.0 AU/mL and purple color indicates values > 0.0 AU/mL. P-value calculated by Wilcoxon rank sum test on the difference in distribution of CXCL13 values between the two groups is indicated on the bracket in the top of the plot (p=0.11). Horizontal bars indicate median values for each group. Antibiotics before LP: 'yes' if patients received at least 1 day of antibiotic treatment during the last 14 days prior to diagnostic lumbar puncture.

c) CXCL13 values in LNB patients by grade of CSF pleocytosis

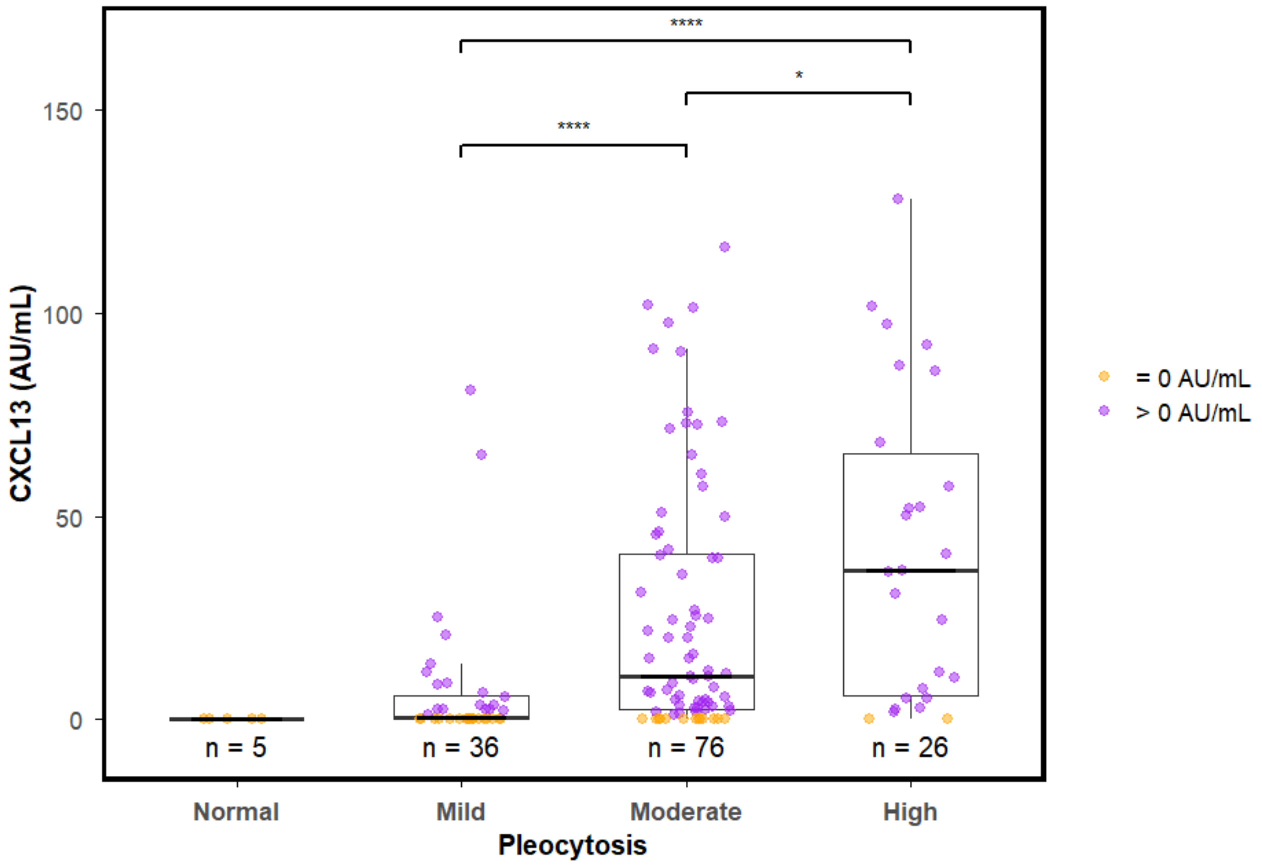

CXCL13= C-X-C motif ligand 13, AU/mL = arbitrary units per milliliter. Orange color indicates CXCL13 values equal to 0.0 AU/mL and purple color indicates values > 0.0 AU/mL. Significance levels indicated by number of stars (*= p <0.05, **= p<0.01, ***=p<0.005, ****=p<0.001) calculated by Wilcoxon rank sum tests as indicated on brackets. Lower borders of boxplots indicate 1st quartile values, bold horizontal lines indicate median values and upper borders indicate 3rd quartile values. Pleocytosis groups: Normal: CSF WBC <5 x10^6 cells/L. Mild: 5-50 x10^6 cells/L. Moderate 50-250 x10^6 cells/L. High: >250 x10^6 cells/L.

d) CXCL13 values in LNB patients by level of CSF protein

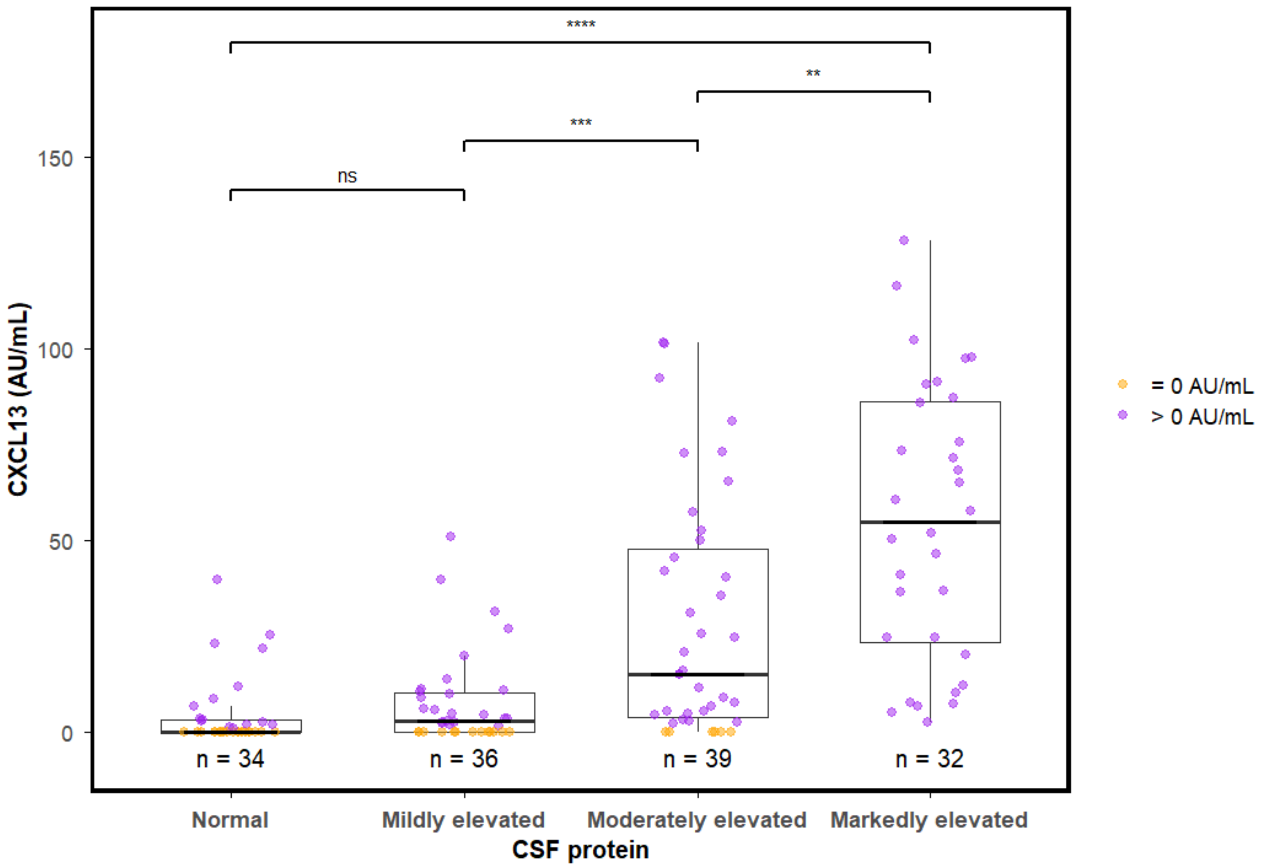

CXCL13= C-X-C motif ligand 13, AU/mL = arbitrary units per milliliter. Orange color indicates CXCL13 values equal to 0.0 AU/mL and purple color indicates values > 0.0 AU/mL. P-values calculated by Wilcoxon rank sum tests are indicated on the brackets in the top of the plot (ns= not significant (p>0.05),*= p <0.05, **= p<0.01, ***=p<0.005, ****=p<0.001). Lower borders of boxplots are 1st quartile values, bold horizontal lines indicate median values and upper borders indicate 3rd quartile values. CSF protein groups: Normal: CSF protein <0.45 g/L. Mildly elevated: 0.45-1.0 g/L. Moderately elevated: 1.0-2.0 g/L. Markedly elevated: >2.0 g/L.
